# Comparison of hydrophilic ophthalmic media on silicone oil emulsification

**DOI:** 10.1371/journal.pone.0235067

**Published:** 2020-06-19

**Authors:** Judit Soós, Miklós D. Resch, Szilvia Berkó, Anita Kovács, Gábor Katona, Andrea Facskó, Erzsébet Csányi, Mária Budai-Szűcs

**Affiliations:** 1 Department of Ophthalmology, University of Szeged, Szeged, Hungary; 2 Doctoral School of Clinical Medicine, University of Szeged, Szeged, Hungary; 3 Department of Ophthalmology, Semmelweis University, Budapest, Hungary; 4 Department of Pharmaceutical Technology and Regulatory Affairs, University of Szeged, Szeged, Hungary; University of New South Wales, AUSTRALIA

## Abstract

The aim of this study was to investigate whether and how the biological media which are in contact with silicone oil play a role in the silicone emulsification process.

Commercially available Oxane 1300 silicone oil and potential hydrophilic phases of the emulsions in the eye (porcine aqueous humor, porcine vitreous and balanced salt solution) were investigated separately and in a mixture or emulsions by means of surface tension, rheological, zeta potential measurements and microscopic investigation.

The surface tension of biological media (vitreous and aqueous humor) was significantly lower than that of non-biological media, especially in the case of aqueous humor, which indicates a remarkable emulsification tendency with these phases. The biological media are able to form both oil-in-water and water-in-oil emulsions, which can be observed in the clinical practice as well. It was established that the vitreous has a more expressed emulsification ability compared with the aqueous humor because smaller and more stable droplets can form with silicon oil when the vitreous is still there. It can be concluded that the vitreous has a higher impact on emulsification than the aqueous medium, which can predict that the vitreous remaining after vitrectomy has a key role in emulsion formation in the eye with silicone oil endotamponade.

## Introduction

Silicone oil endotamponade has frequently been applied in the last decades since its introduction by Armaly [1] and Cibis in 1962 [2]. After implantation, silicone oil forms a spherical bubble in the vitreous cavity, but some residual vitreous can remain at the vitreous base, especially in phakic patients. Intraocular silicone oil is in contact with the residual vitreous, and aqueous humor secreted by the ciliary body. Silicone oil, however, may undergo emulsification in 4–72% of the cases and lead to vision-threatening complications affecting nearly all ocular structures. Complications can include corneal decompensation, band keratopathy, acute and chronic changes in intraocular pressure, lens opacities, epiretinal membrane, retinopathy, optic neuropathy, and extraocular extension (such as migration into the optic nerve, chiasm and even into the cerebral ventricular system) [3–8]. The emulsification of silicone oil depends on the interfacial tension between the oil and the hydrophilic phases (aqueous, vitreous or balanced salt solution (BSS)), which means decreased interfacial tension results in increasing emulsification tendency. The time course and level of emulsification are rather variable [9], but it was established to occur within the first year and mainly after the 5^th^ postoperative month [10].

Besides the physicochemical characteristics of silicone oils, biological factors such as biological environment, emulsifiers and mechanical effects can influence the development of emulsification. On the other hand, based on literature information, to date there is no data on how the complex ocular biological media can influence emulsification.

The purpose of the current study was to develop an in vitro model for the complex investigation of the phenomenon of silicone oil emulsification in the presence of potential ophthalmic hydrophilic phases such as aqueous humor, vitreous and BSS. Our aim was to evaluate the emulsification ability of the biological media and to analyze the formed emulsions.

## Materials and methods

### Materials

#### Silicone oil

Original silicone oil Oxane 1300 (Bausch&Lomb GmbH, Germany) (SiO) and potential hydrophilic and lipophilic phases of the emulsions were investigated separately and in a mixture or emulsions.

#### Hydrophilic phases

For the hydrophilic phase of the emulsions balanced salt solution (BSS, Alcon Laboratories, Inc., USA) and biological aqueous phases such as porcine aqueous humor (AH) and porcine vitreous (VB) were used. Porcine eyes were freshly obtained from a slaughterhouse (Pick Szeged Zrt., Szeged, Hungary) (animals were not sacrificed for the study). The aqueous was aspirated through corneal paracentesis and the vitreous was removed with scissors 2–5 hours postmortem from 100 porcine eyes. The humors were homogenized, chilled and stored at -20°C. They were melted at room temperature just before the application. Our applied microscopical and zeta potential methods required high optical purity, but the biological fluids contain often pigments and other non-soluble components. Based on our preformulation studies, membrane filters with pore size 0.45 and 0.20 microns were not suitable for the separation of the non-soluble components. Therefore the clear biological aqueous phases were separated from the pigments by centrifugation in Vivaspin 15R 5,000 MWCO Hydrosart tubes (Sartorius, Stonehouse, UK) with Hermle Z323K, (HERMLE Labortechnik GmbH, Wehingen, Germany). Centrifugation was performed at 5000 rpm (9000 rcf), for 30 min at 4°C. This separation method resulted optically clear biological fluids. Mixtures of the vitreous and BSS were made to model *in vivo* vitrectomized (and silicone oil filled) eyes with residual vitreous.

#### In vitro emulsions

Silicone oil and the hydrophilic phases were mixed with a Vortex stirrer at 50% of maximum speed for 5 min. The emulsions/mixtures were measured immediately after preparation. Each emulsion was prepared three times. Two ratios of the oil and aqueous phase were applied 2:8 and 8:2.

### Methods

#### Surface tension

Original silicone oils and aqueous phases were analyzed by means of surface tension measurements, by OCA 20 instrument (Dataphysics Instruments GmbH, Filderstadt, Germany) using the pendant drop method. The instrument detects the contour of the pendant drop and calculates the surface tension. The mean of ten parallel measurements was used for calculations.

#### Microscopic investigation

For the microscopic observations, the hydrophilic phases of the emulsions were stained with 0.001% Fluorescein sodium (Sigma-Aldrich GmbH, Germany) in a 100:1 ratio. 50 μL of emulsions were put onto microscope slide and covered with cover glass. Microscopic images were made by a Leica DMB6 microscope (Biomarker GmBH, Hungary) in fluorescence mode (Leica DFC7000 T fluorescence camera, Biomarker GmBH, Hungary). The magnification was 200x. Droplet size and droplet size distribution were analyzed on the basis of the black and green contour of the oily and aqueous phase using the LAS X core software of the microscope.

Each composition was prepared three times, and each sample was scanned by microscope. The images, where droplets were observed, were analyzed by the software.

Emulsions with both ratios (2:8 and 8:2 oil and aqueous phase ratios) were also investigated.

#### Zeta potential

The zeta potential of the in vitro emulsion was measured by Zetasizer Nano ZS (Malvern Instrument, UK) with electrophoretic light scattering. Emulsion droplets are surrounded by an electric double layer. This double layer is formed when surface-charge-carrying emulsion droplets are surrounded by counter-ions of charge opposite to that of the droplet surface. A given amount of these counter-ions move together with the droplet, hence a slipping plane beyond the emulsion droplet. The electrical potential at this slipping plane is the zeta potential (ζ, mV). As a result of increased electrostatic repulsion, the coalescence of the oil droplets is hindered. An emulsion with a high absolute value of zeta potential is more stable in comparison to that with lower zeta potential absolute values [11].

With this method, emulsions with 2:8 oil and aqueous phase ratio were investigated.

#### Rheology

The viscosity of silicone oil and *in vitro* formed emulsions/mixtures was measured by a Physica MCR 101 rheometer (Anton Paar, Austria). The measuring device was cone and plate type (diameter 25 mm, gap height in the middle of the cone 0.046 mm, cone angle 1°). The flow and the viscosity curves of the samples were plotted. The shear rate was changed from 0.1 to 100 s^-1^. The viscosity of the samples was evaluated at 100 s^-1^. The experiments were carried out in triplicate.

With this method, emulsions with 2:8 oil and aqueous phase ratio were investigated.

### Statistics

Unpaired *t*-test was performed using GraphPad Prism (GraphPad Software Inc., USA). P value < 0.05 was considered to be statistically significant. A level of p ≤ 0.05 was taken as significant, p ≤ 0.01 as very significant, and p ≤ 0.001 as highly significant.

## Results

### Surface tension

**Fig 1:**
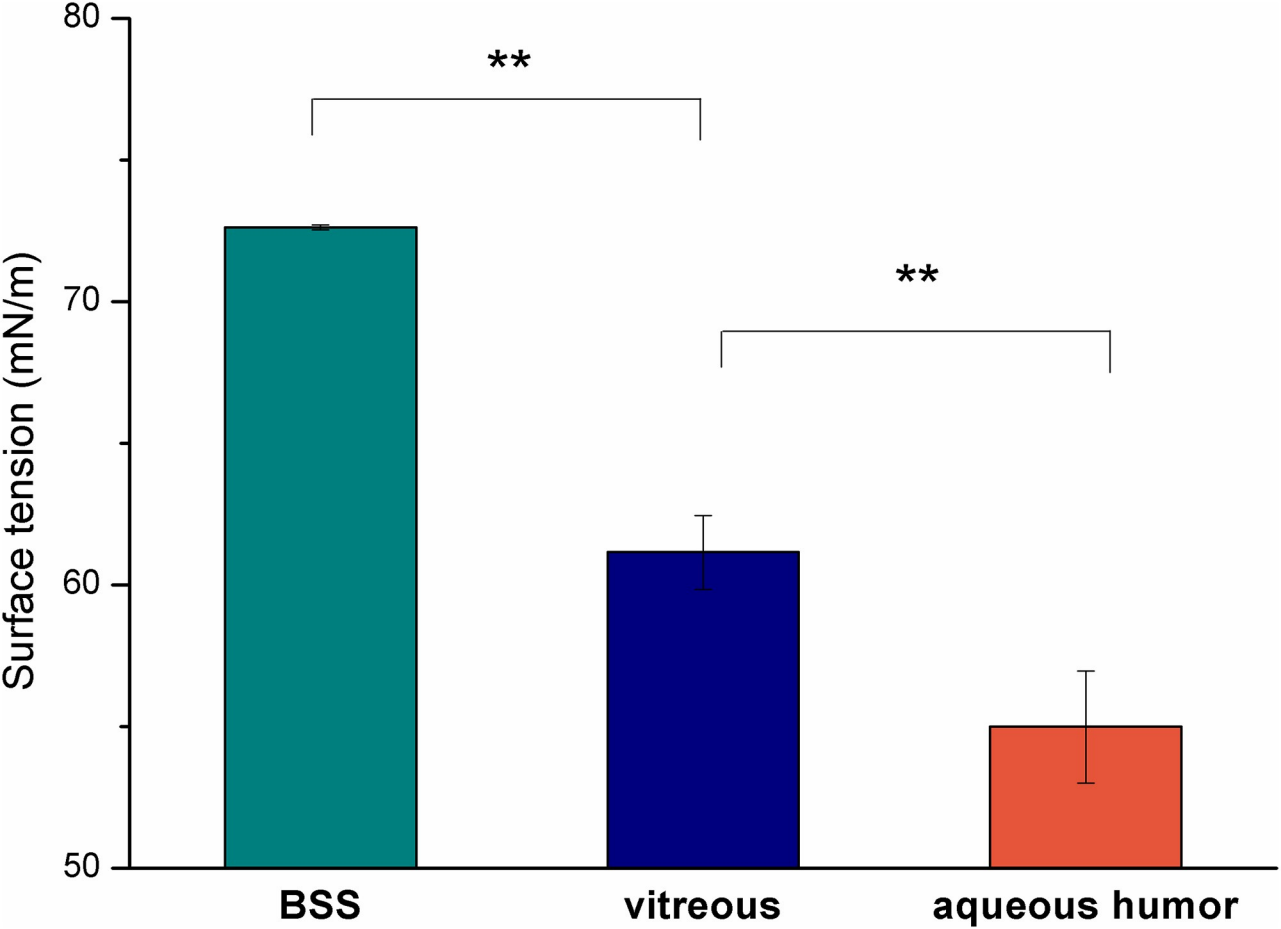
Surface tension of the aqueous phases (** p<0.01).

All hydrophilic substances had significantly lower surface tension than that of BSS. Aqueous humor had the lowest surface tension (54.99 ± 1.98 mN/m), which was followed by the value of the vitreous (61.16 ± 1.30 mN/m), while BSS had the highest value (72.62 ± 0.08 mN/m) (Fig 1). The lower surface tension of the hydrophilic phase can mean lower interfacial tension.

The surface tension of fresh and freeze-thaw vitreous was also compared in order to investigate the effect of the freezing. Our results indicated the surface tension remained the same (S1 Fig).

### Microscopic investigation

**Fig 2:**
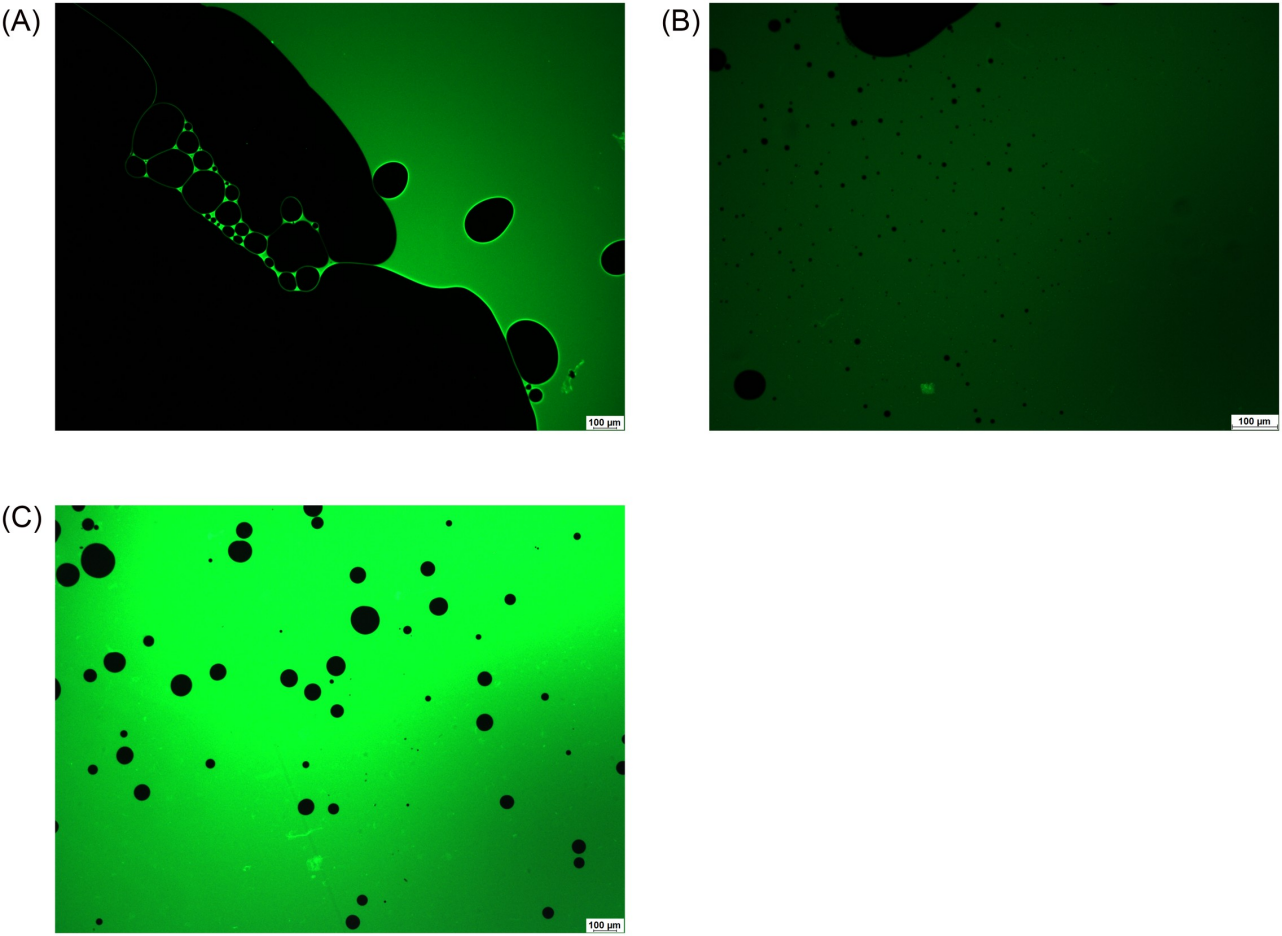
Microscopic pictures of emulsions containing BSS (a), VB (b) or AH (c) in 2:8 oil and aqueous phase ratio.

For easy distinction, a fluorescent dye was applied during the microscopic measurements. Green fluorescein sodium can dye the aqueous phases of the emulsions, while silicone oil can be seen with black color in the pictures. A few large and many small black oil droplets can be observed in the green aqueous phase in the microscopic images (Fig 2a, 2b and 2c) when a high amount of aqueous phase (80%) was applied. At this oil:water ratio an oil-in-water type emulsion was formed.

The microscopic image of the emulsion containing VB showed a greater amount and smaller oil droplets in the aqueous phase compared with that of the emulsion with AH, while in the case of BSS the typical picture of the emulsion was a main giant continuous oil droplet and a few bigger ones (Fig 2a).

**Fig 3:**
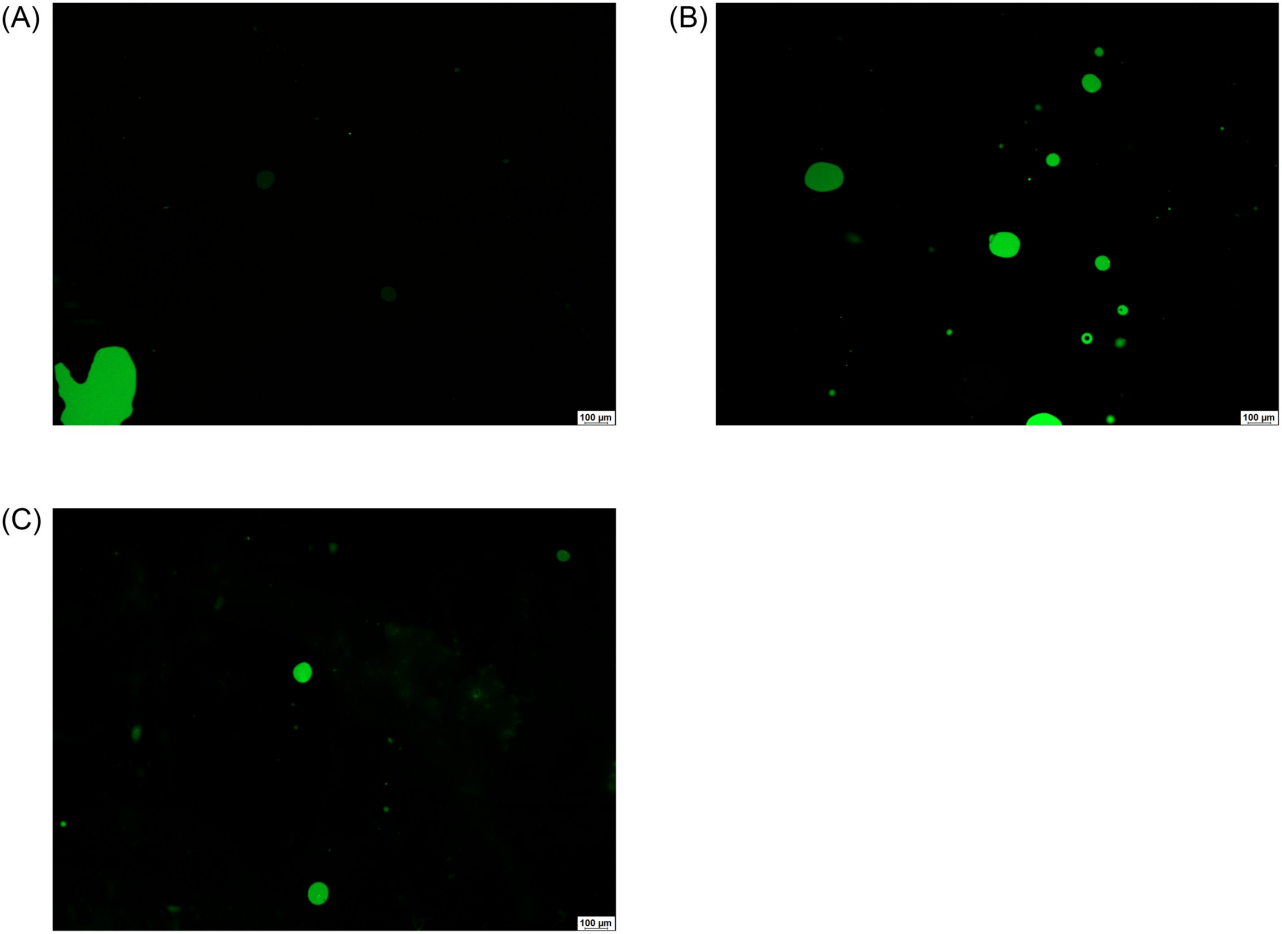
Microscopic pictures of emulsions containing BSS (a), VB (b) or AH (c in 8:2 oil and aqueous phase ratio.

When a higher amount of silicone oil (80%) was applied, a water-in-oil type emulsion could be observed (small green aqueous droplets in the black oil phase) (Fig 3a, 3b and 3c). There was no difference between the two emulsions containing biological media, while the droplet frequency was lower when BSS was used as the hydrophilic phase of the emulsions.

**Fig 4:**
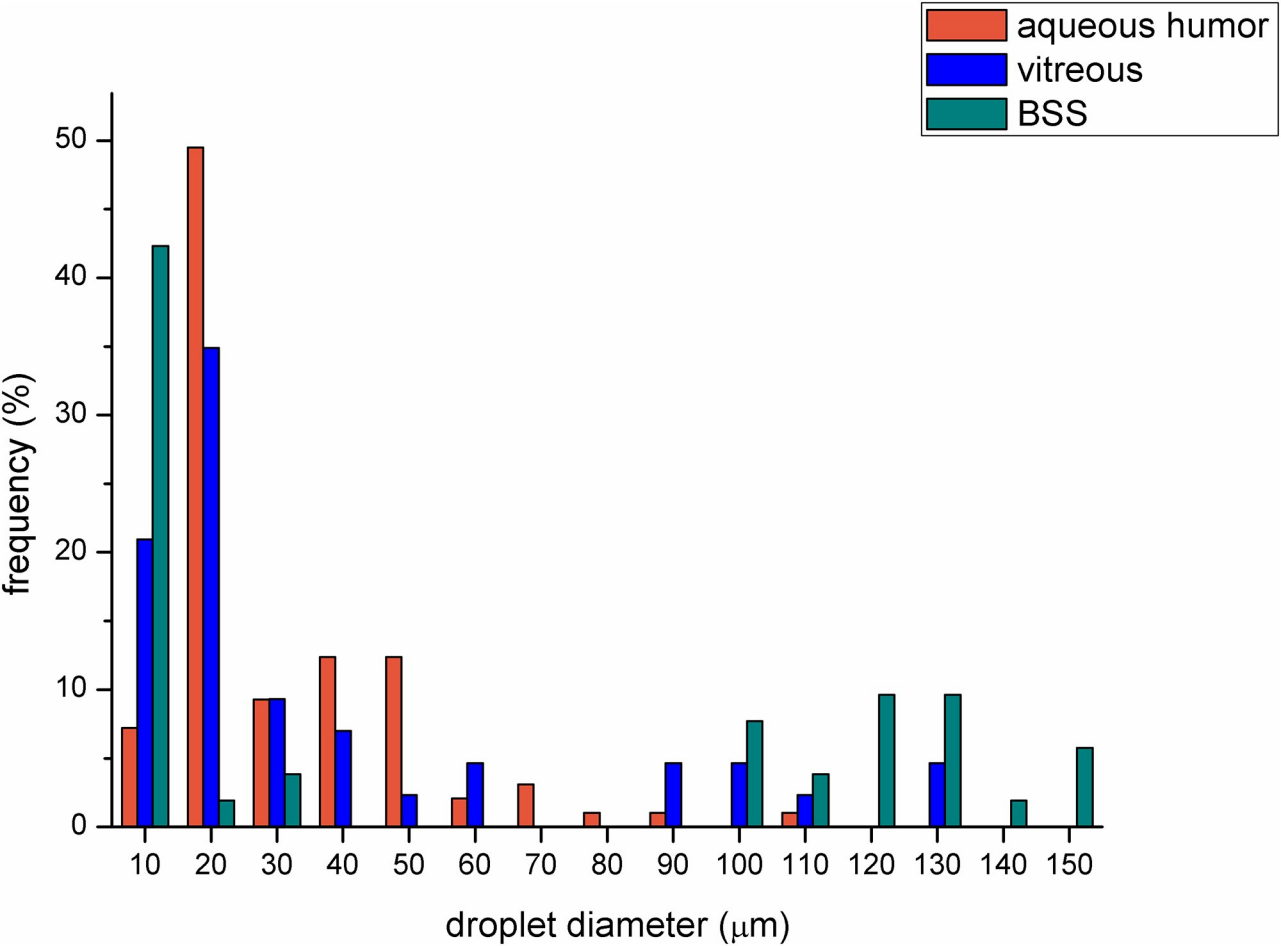
Droplet size distribution of emulsions containing different aqueous media in 2:8 oil and aqueous phase ratio (the number of analyzed droplet for BSS, VB and AH compositions were 52, 43 and 97, respectively).

The sharp contour of the droplets made it possible to analyze droplet size and droplet size distribution using the software of the microscope (Fig 4). The emulsions containing BSS proved to have greater droplet frequency at bigger droplet size (above 100 μm). The droplet size distributions of the emulsions containing VB or AH were almost similar, but in the case of VB a higher amount of smaller droplets could be observed (more remarkable frequency below 20 μm).

### Droplet size and zeta potential

Zetasizer was used to measure droplet size from nanometer to several microns using dynamic light scattering, and zeta potential using electrophoretic light scattering. This type of droplet size measurement can avoid the errors from microscope resolution and from the selection of the photo imaging.

**Fig 5:**
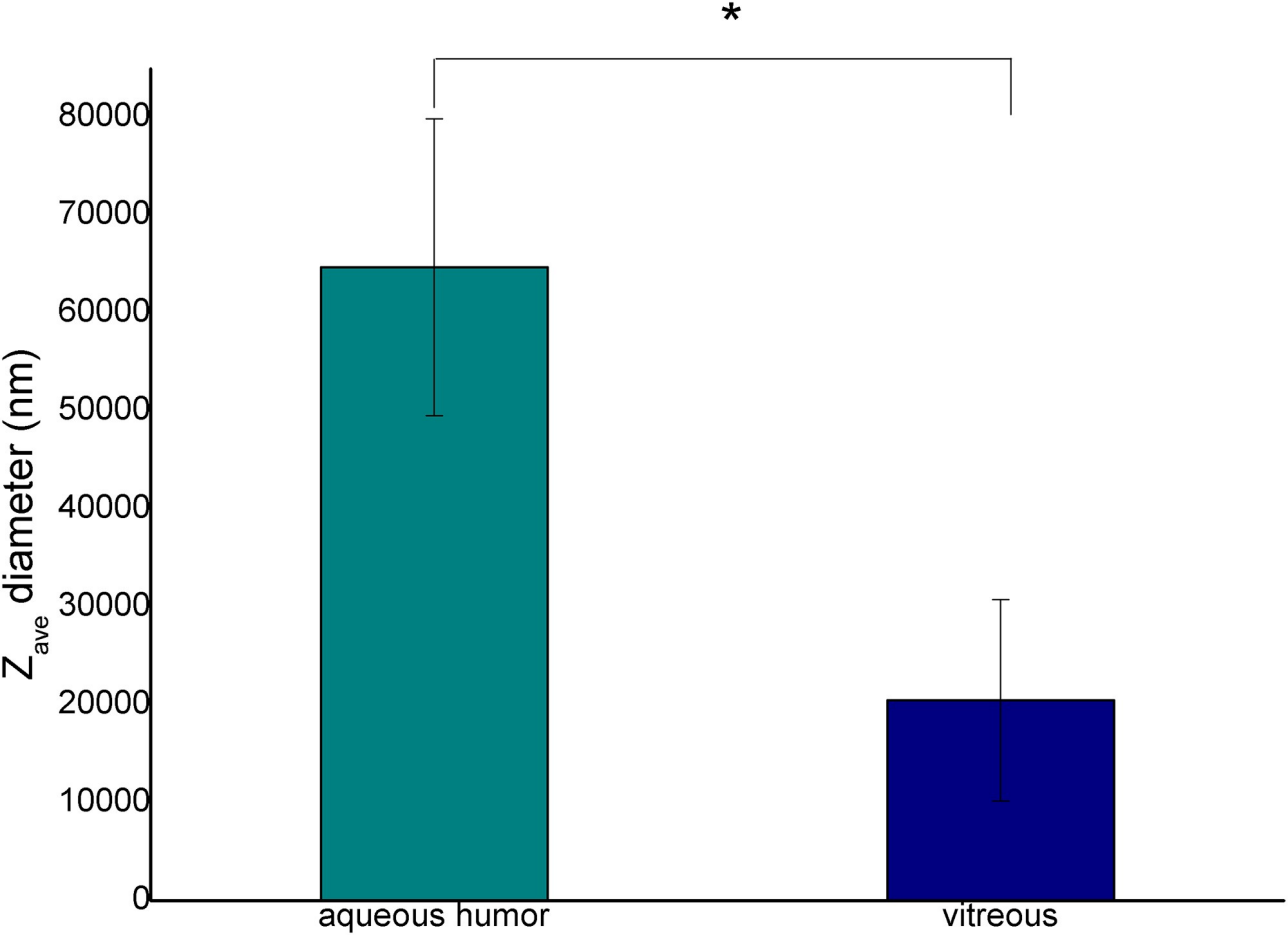
Diameter of the emulsion droplets at 2:8 oil and aqueous phase ratio (* p ≤ 0.05).

In our work the droplet size and the zeta potentials of emulsions containing 20% silicone oil were only measured (Fig 5 and S1 Table), since a larger percent of oil could not be measured by this method.

The average droplet size (Z_ave_) of emulsions containing VB was significantly smaller (about 20 μm) (p = 0.018), while that of emulsions containing AH was bigger (over 60 μm). This observation correlates with the results of the droplet size distribution presented in the microscopic investigations (Fig 4).

**Fig 6:**
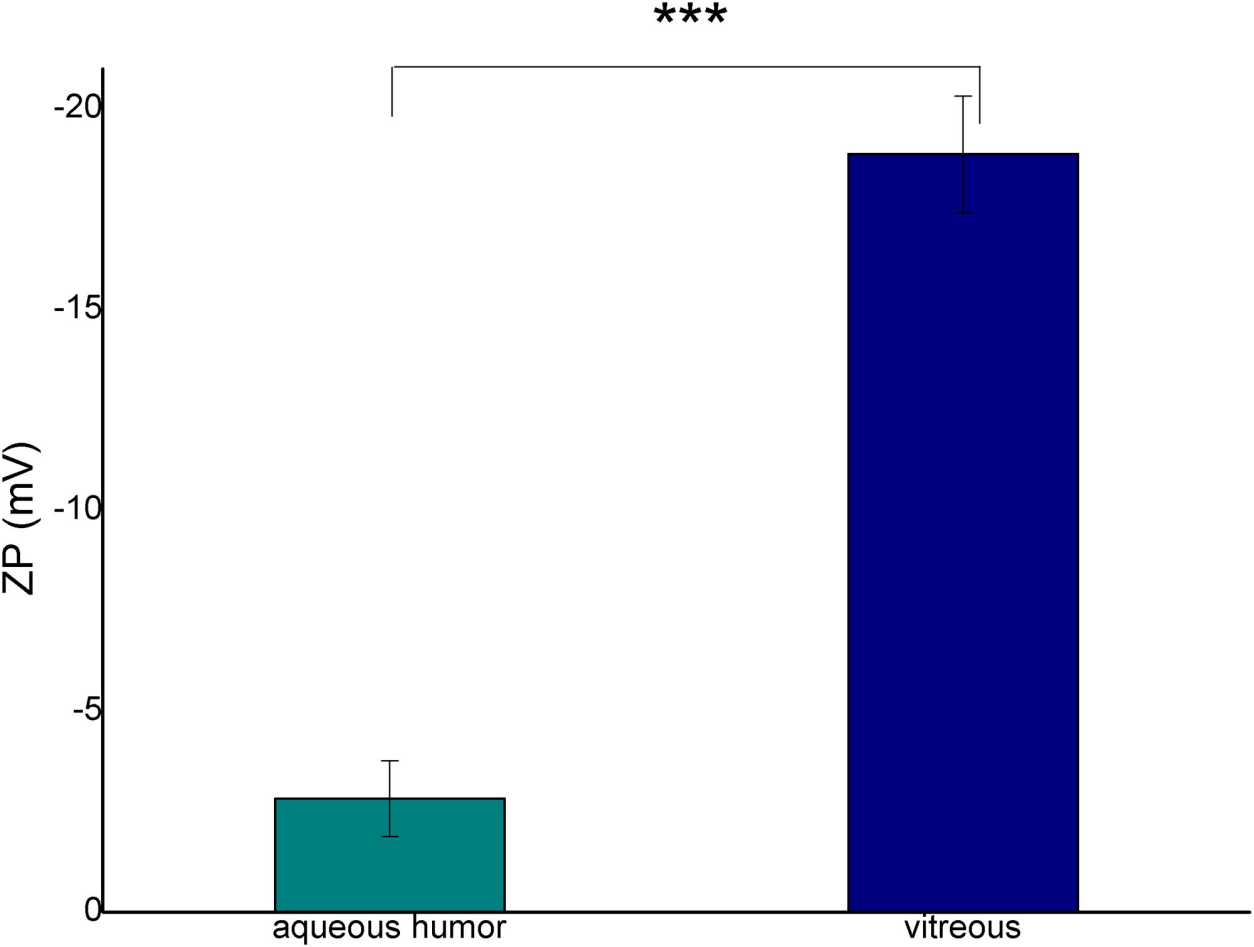
Zeta potential of the emulsions at 2:8 oil and aqueous phase ratio (*** p ≤ 0.001).

The absolute zeta potential of the emulsion with VB was remarkably higher than that of AH (Fig 6). The zeta potential value can indicate the formation of an electric double layer surrounding the oil droplets stabilizing the emulsion droplets. The increase of the absolute values can predict an improvement in the stability of the disperse system.

### Rheology

The viscosity of the original oil Oxane 1300 was measured to be 1313.3±5.8 mPa*s (mean±SD). Emulsions were prepared on the basis of the “In vitro emulsion section”, where the Oxane concentration was 80%, while the aqueous phase (AH or VB) concentration was 20%. Each emulsion was prepared in triplicate.

In the case of emulsions containing AH, the viscosity values were lower than those of Oxane, and the standard deviation (SD) was very high. Contrarily, the emulsions containing VB revealed elevated viscosity values with moderate standard deviation (Table 1.)

**Table 1:** Viscosity value of Oxane 1300 and the emulsions in 2:8 oil and aqueous phase ratio.

	Viscosity (mPa*s)				
	1	2	3	Mean	SD
**Oxane 1300**	1310	1320	1310	1313.3	5.8
**Emulsion with AH**	1000	702	65	589.0	477.6
**Emulsion with VB**	1490	1540	1320	1450.0	115.3

If an inhomogeneous system is formed (not a stable emulsion system), it will result in varying viscosity data, as we can see for emulsions containing AH. When an emulsion is formed (aqueous droplet in the oily phase), a higher viscosity value can be measured due to the viscosity increasing effect of the dispersed droplets (this tendency can be observed in the case of emulsions with VB) [12].

## Discussion

The implanted silicone oil forms a giant continuous oil droplet in the vitreous cavity, which is surrounded by the thin layer of the hydrophilic phase remaining in the eye. This hydrophilic phase can be a leftover vitreous, aqueous humor secreted by the ciliary body, or buffer solution applied during vitrectomy. Silicone oils produce high interfacial tension opposite to the hydrophilic phases, and this behavior enables these materials to exert an intraocular endotamponade effect in the vitreous body. Despite the high surface tension, in practice silicone oils undergo emulsification, which can lead to several complications including cataract, keratopathy, glaucoma and silicone retinopathy [13]. Silicone oils have excellent chemical stability, but many studies proved that they are not inert biologically, some impurities such as retinol [14], low-molecular-weight components (LMWC) [4], residual catalysts [15], OH-endgroups [16], and cholesterol [17] were detected in silicone oils, which can probably improve emulsification due to the modification of the surface tension of the oil. The other possible cause of emulsification is the mechanical effect, therefore, some literature analyzed the effect of different agitators and eye movement on emulsification using special eye chambers, which mimic the eye movement in vitro [18, 19].

A new mechanism of SiO emulsification was also proposed, where break-ups of SiO lead to its adherence to ocular tissue and thus forming emulsified droplets. This mechanism can decrease with the application of high-molecular-weight SiOs [20, 21] and preactivated SiOs, the latter is able to form a cohesive gel in the vitreous cavity [22].

Impurities and mechanical effects can be largely patient-dependent because these factors can be disease or surgical action related. In our work we focused on different biological and surgical hydrophilic phases which can be present during vitrectomy and can be responsible for the early development of emulsification. These hydrophilic phases can be in contact with silicone oil and are able to form an interface. When the surface tension at the interface is low and a mechanical effect occurs, it promotes the formation of droplets. In our work the surface tensions of the possible hydrophilic phase were investigated, and it was found that the biological media have significantly lower surface tension compared to the non-biological ones (BSS) (about 61 and 54 mN/m compared with 72 mN/m). On the basis of Antonov’s rule and its later corrections and modifications [23], these lower surface tension values can mean lower interfacial tension between silicone oils and biological aqueous media, and the lower interfacial tension can result in increased emulsification.

In our work we formulated in vitro emulsions with silicone oil and the possible hydrophilic phases in order to investigate the structure and stability of the emulsions, and also to compare the emulsification ability of the different hydrophilic phases.

Two types of emulsion can develop after silicone oil implantation, both of them can be observed in clinical practice. In the case of the first emulsion type, the oil droplets get loose from the giant continuous oil droplet and form an oil-in-water emulsion (o/w emulsion). These emulsified oil droplets are able to move easily in the hydrophilic phase, thus they can be present in the aqueous humor, which can be seen in the anterior chamber in the human eye after undergoing vitrectomy with silicone oil endotamponade. On the other hand, the migration of small oil droplets can lead to secondary silicomacrophagocytic open-angle glaucoma [24]. In our study this type of emulsion could be observed when we applied a high amount of biological hydrophilic phase. It had already been established in earlier studies that the components of the biological medium have a strong effect on emulsification. The role of fibrinogen, fibrin, γ-globulin, VLDL, alfa-1-glycoprotein in emulsification had already been clarified in vitro [16]. In our work the complex hydrophilic phases were compared concerning the emulsification tendency. The aqueous phases were indicated by fluorescent dye, thus black oil droplets were perceptible in the green aqueous phase on the microscope slide of our in vitro emulsions (Fig 2). The number of the oil droplets was higher, and the droplet size was smaller in the case of VB compared with the BSS and AH containing systems. This observation was confirmed by the droplet size and zeta potential measurements. Zeta potential is a sensitive indicator of the stability of disperse systems such as emulsions. Higher absolute Zeta potential means an effective stabilizing layer around the droplets, which hinders the adhesion and the fusion of the droplets. In contrast with microscopic investigation, where the presented and analyzed slides are chosen by the investigator, these types of measurements are more investigator-independent, a bigger amount of the sample can be analyzed using dynamic and electrophoretic light scattering, which results in more representative data from the sample. In this measurement, similarly to the microscopic investigation, smaller oil droplets were present in the case of VB, furthermore the absolute Zeta potential of the emulsions containing VB was also higher compared with emulsions containing AH. These statements can mean that the residual vitreous body, which can form an interface with the silicone oil droplet, has more emulsification potential, and it can be the starting point of further emulsification. On the other hand, the formed oil droplets are more stable in the vitreous (indicated by the higher absolute Zeta potential value), predicting that the oil droplet will remain and will not fuse with the continuous oil droplet later.

Earlier studies established the role of the characteristics of the silicone oil and the surface-active agents in the biological environment in the emulsification. The presence of the very small amounts of emulsifier can facilitates the formation of an emulsion. Various biologic surface-active agent can accumulate in hemorrhagic and inflammatory situations in the aqueous biological media such as fibrin, fibrinogen, gamma globulins, acidic alpha 1-glycoprotein, very low-density lipoprotein [10]. In our present biological media were collected from healthy animals and were investigated as a complex fluid. We cannot denominate only one two or three components whose concentration or presence can be the key factor(s) in the emulsification, but we can clearly see, the normal biological fluids have remarkable emulsifying effect. In addition, it is also clear from the results that the residual vitreous can have a much greater effect on subsequent emulsification than aqueous.

As for the second emulsion type, droplets from the hydrophilic phase can form and enter the oil phase resulting in a water-in-oil type emulsion (w/o emulsion). This type of emulsion can be observed in the vitreous cavity when the opalescence of silicon oil is noticed in clinical practice. In our study this type of emulsion was obtained when lower hydrophilic phase was applied (20%). The microscopic investigation indicated hydrophilic droplets in the continuous oil phase in all cases, but the biological media showed remarkable emulsification tendency. In order to compare the emulsification ability of the two biological media as a water-in-oil emulsion, rheological investigation was performed and the viscosity of the systems was evaluated. This method is considered to be a more investigator-independent test than the zeta potential measurement. In our work the viscosities of the w/o emulsions containing biological media were compared with the original oil viscosity data. When a nearly stable w/o emulsion is formed, the emulsified droplets increase the viscosity of the continuous phase, in our case this phase is silicone oil. Considering the viscosity data of the formulated in vitro w/o emulsions, mixtures with AH exhibited varying viscosity data indicating an unstable system, while the viscosity of the emulsion containing VB showed an elevated value, which predicts a real, stable emulsion formation. The latter suggests the VB droplets are more stable in the continuous silicone phase.

## Conclusions

As a practical conclusion, the residual vitreous body can increase the risk of the emulsification of silicone oil because it has remarkable emulsification potential concerning both emulsion types (w/o and o/w). The amount of the residual can be also critical because a higher amount can form a larger interface between the oil and the vitreous, and it can result in increasing the risk of starting the emulsification. Our multimodal complex in vitro model provided evidence on the clinically known feature, namely that as complete a removal of the vitreous should be performed as possible in order to minimize the emulsification ability of biological media.

## Supporting information

S1 Fig Surface tension of fresh and freeze-thaw vitreous. (TIF)

S1 Table Z_ave_ and ZP values of the emulsions. (DOCX)
