# Supplementary material for: Comparison of hydrophilic ophthalmic media on silicone oil emulsification
Source: PLoS One. 2020 Jun 19;15(6):e0235067. doi: 10.1371/journal.pone.0235067 (PMC7304586; doi:10.1371/journal.pone.0235067)
Supplement: S1 Table — (DOCX) [file pone.0235067.s002.docx]

**S1 Table. Z_ave_ and ZP values of the emulsions.**

|  | Z_ave_ diameter (nm) | | | | | ZP (mV) | | | | |
| --- | --- | --- | --- | --- | --- | --- | --- | --- | --- | --- |
|  | 1 | 2 | 3 | Mean | SD | 1 | 2 | 3 | Mean | SD |
| AH | 47210 | 72950 | 73990 | 64717 | ±15170 | -1.93 | -2.74 | -3.81 | -2.83 | ±0.95 |
| VB | 24030 | 8870 | 28500 | 20467 | ±10289 | -19.6 | -19.8 | -17.2 | -18.87 | ±1.45 |
